# Polypropylene Blend with Polyphenols through Dynamic Vulcanization: Mechanical, Rheological, Crystalline, Thermal, and UV Protective Property

**DOI:** 10.3390/polym11071108

**Published:** 2019-07-01

**Authors:** Jingjing Liao, Nicolas Brosse, Antonio Pizzi, Sandrine Hoppe, Xuedong Xi, Xiaojian Zhou

**Affiliations:** 1 LERMAB, University of Lorraine, Boulevard des Aiguillettes BP 70239, 54506 Vandœuvre-lès-Nancy, France; 2 Key Laboratory for Forest Resources Conservation and Utilisation in the Southwest Mountains of China (Southwest Forestry University), Ministry of Education, Kunming 650224, China; 3 LRGP, University of Lorraine, 1, Rue Grandville, BP 451, 54001 Nancy Cedex, France

**Keywords:** tannin, lignin, dynamic vulcanization, polypropylene, UV weathering

## Abstract

Tannin and lignin were blended with polypropylene (PP) through dynamic vulcanization technique. Their influence of mechanical property, crystallinity, thermal stability, as well as ultraviolet (UV) protection property on the PP matrix was investigated and compared with native tannin and lignin. According to our experimental results, tannin and lignin undergo dynamic vulcanization and were more compatible with the PP matrix. Besides, tannin and vulcanized tannin can perform as nucleating agents of PP because of their relatively small particle size. Moreover, vulcanized tannin/lignin have a better performance on the thermal stability of PP compared with native tannin/lignin, especially PP/vulcanized lignin blend. Furthermore, vulcanized tannin/lignin present better UV protective performance, concluded from fewer changes on surface morphology, carbonyl index, crystallinity, viscosity, and tensile property.

## 1. Introduction

**Figure 1:**
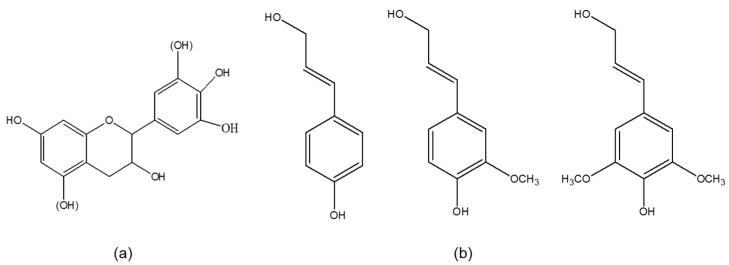
Structure of flavonoid unit of condensed tannins (**a**) and phenylpropanoid units of lignin (**b**).

Polypropylene (PP) is a typical semi-crystalline thermoplastic derived from the olefin monomer propylene. Since being discovered in 1954, PP has quickly become one of the most popular commodity plastics due to its low density and low cost with good mechanical property [1]. Today, PP is widely used as a composite matrix to process composite materials because of the excellent chemical resistance; hence, it can be easily processed by many efficient converting methods, like injection molding and extrusion [2]. The combination of PP with polymers from sustainable resources has been extensively studied over the past decades in order to reduce the dependence on petrochemical-based plastics [3]. Generally, composite materials based on PP and biopolymers have a lower lightweight and less environment impact [4]. Apart from these major advantages, biopolymers bear individual structural features associated with specific properties; hence, they are potential functional elements for PP. For example, cellulose is generally used as a reinforcement of PP [5]; starch and polylactic acid can improve the degradability of PP [6]; carbohydrates, proteins, lipids, and phenolic compounds can be flame retardants of PP [7]. Among these biopolymers, phenolic compounds (e.g., lignin and tannin) incorporated with PP have attracted interest for both academia and industry, because PP is highly sensitive to UV light and oxygen compared with other polyolefins, while phenolic compounds can prevent its thermo- and photo-degradation, owing to their chemical structure. As shown in Figure 1, flavonoid structure of tannin (Figure 1a) [8] and the phenylpropanoid (C9) units of lignin (Figure 1b) [9] are inherent hindered phenolic structures, which can stabilize themselves into stable phenoxy radicals, preventing initiation of new radicals [10]. 

Lignin is the most abundant phenolic compound on the earth and is a widely distributed biopolymer. Lignin is usually viewed as a waste in the pulp and paper industry [11]. According to the published reports, lignin can be a potential precursor for synthesized thermoset resin, like polyurethanes [12], epoxy resins [13], and wood adhesives [14]. Moreover, lignin has been considered as a potential functional element for polymer composites in the past decades and has been included as a compatibilizer [15], antioxidant [16,17], stabilizer [18,19], and reinforcement [20]. However, the large molecular weight and hydrophilic characteristic of lignin lead to low reactivity and poor compatibility with the hydrophobic polymer matrix and would result in the deterioration of the polymer’s mechanical properties [21,22]. To solve this problem, chemically modified lignins (e.g., esterification, etherification, polymer grafting) have been examined to enhance the compatibility with polymers [22]. However, esterification significantly decreased the phenolic content of lignin and consequently decreased the antioxidant effect [21].

Tannins are widely present in soft tissues of woody plants like leaves, needles, and bark. They are easily extracted using water or organic solvent, include methanol, ethanol, and acetone [23]. Tannins have been extensively described for the production of green wood adhesives, which displayed properties comparable to those of conventional phenolic adhesives [23]. Unlike lignin, the research about tannins-based thermoplastic composites are rare. Published studies focus on the preparation of thermoset tannin resins, namely polyurethane foam material [24] and thermoset plastic [25,26,27]. Tannins used as an additive at low content have been reported as a good stabilizer with antioxidant and UV-protective properties on polypropylene [28,29], polyethylene [30], poly(vinyl chloride) (PVC) [31], and polyvinyl alcohol [32]. The chemical modifications of tannin have been investigated to improve its compatibility with the polymer matrix. For example, esterified tannin [33] exhibited improved miscibility with hydrophobic plastic matrix (e.g., poly(butylene succinate) [34], PP [35], aliphatic polyester [35], poly(lactic acid) [36,37]). In addition, it has been shown that low esterified tannin displayed UV stability, and tannin with long ester chain length exhibited plasticity function. According to the report of Bridson [38] and co-workers, PLA had the possibility to incorporate a high content of hydroxypropylated tannin through trans-esterification reactions; this totally bio-composite material has revealed potential as a precursor for carbon material. Besides, another research reported PVC incorporated with epoxied tannin, that can enhance dynamic thermal and process stability, and improve the rheological properties on PVC [39]. 

Dynamic vulcanization is an traditional technique to prepare thermoplastic-elastomer composites [40], with which an elastomer can be vulcanized (crosslinked) simultaneously during its mixing with a thermoplastic polymer. Similarly, thermoset resin can be crosslinked during the extrusion process and simultaneously dispersed in the thermoplastic matrix. Few researchers reported the successful utilization of dynamic vulcanization of PP/thermoset resin system [41,42,43,44] like epoxy and novolac. These experimental results exhibited higher modulus, stiffness, as well as thermal stability compared to neat PP. 

In our recent work, we reported for the first time the production of PP/tannin resin composites through dynamic vulcanization [45]. According to our experimental results, tannin was crosslinked by hexamine, forming thermoset particles, well dispersed in PP matrix. The final product displayed higher Young’s modulus and good UV protection capacity. However, even if tannin is well known for its antioxidant property, the effect of the crosslinking process on its UV protection capacity is still unclear. Besides, it should be interesting to use dynamic vulcanization with PP/lignin blends since lignin is widely studied and a low-cost polyphenol. For this purpose, in our current work, native tannin/lignin and crosslinked tannin/lignin were blended with PP, respectively. The influence of phenolics on the mechanical property, crystallinity, thermal stability, as well as UV protection property of PP matrix was investigated. 

## 2. Materials and Methods

### 2.1. Materials

The PP was supplied by TOTAL (Total Research & Technology Feluy, Feluy, Belgium) with a melt flow index of 25 g/min according to the standard test method ISO 3146 and a melting temperature of 165 °C. Mimosa tannin was purchased from Silva Chimica, in Mondovi, Italy. Biochoice kraft lignin supplied by Domtar Inc. (Montreal, QC, Canada). The mimosa tannin and Kraft lignin used have been previously characterized. The mimosa tannin was determined by matrix-assisted laser desorption/ionization time-of-flight (MALDI-TOF); molecular weight distribution ranged from 375 to 2333 Da [46]. Biochoice Kraft lignin were characterized by gel permeation chromatography (GPC). The average molecular weight and molecular weight distribution were *M*_n_ = 949 g/mol, *M*_w_ = 6772 g/mol, and *M*_w_/*M*_n_ = 7.1 [47]. The paraformaldehyde and sodium hydroxide were purchased at Acros Organics (Illkirch-Graffenstaden, France) and VWR company (Radnor, PA, USA). The hexamethylenetetramine 99% (hexamine) and glyoxal (40 wt % solution in H_2_O) were purchased at Acros organics (Illkirch-Graffenstaden, France). All the chemicals were used without further purification.

### 2.2. Pre-Reacted Tannin and Lignin with Glyoxal

In our last work [45], the preparation of tannin-hexamine pre-reactors has been described in detail. The same pathway was applied to prepare tannin-glyoxal pre-reactors.

The lignin-glyoxal pre-reactor was prepared as follow: one hundred parts of Kraft lignin were dissolved to one hundred parts of water. Sodium hydroxide solution (30 wt %) was used to keep the pH of the solution between 12 and 12.5 for better dissolution of lignin. One hundred parts of glyoxal were added, and the lignin solution was then continuously stirred with a magnetic stirrer/hot plate for 30 min. All the compounds were added based on the weight of the dry lignin. 

### 2.3. Spray Dried the Pre-Reacted Tannin and Lignin

The pre-reacted samples were dissolved with 500 parts of water for spray drying. The parameters of a spray dryer (BUCHI mini spray dryer B-290, Flawil, Switzerland) were described elsewhere, tabulated in Table 1:

### 2.4. Composites Preparation

The PP powder was, respectively, well blended with tannin (T), lignin (L), pre-reacted tannin-glyoxal (TG), and lignin-glyoxal (LG) in 90/10 weight proportions in a baker. All blended mixtures were carried out separately in a twin-screw extruder (Thermo Scientific^TM^ Process 11, Villebon-sur-Yvette, France) with a screw rotation speed of 200 rpm at 190 °C. The extruder had a screw diameter of 11 mm and a length-to-diameter ratio L/D = 40. The screw profile is composed of three mixing zones with different configurations of kneading blocks for promoting dispersive and distributive mixing. The residence time was approximate 2 min 30 s. The pre-reacted tannin and lignin were expected to be cured under the pressure and temperature of the extruder. Unreacted species and reaction by-products were removed by a highly efficient vacuum. Those extruded composites were noted as PPT, PPL, PPTG, and PPLG, respectively. PP was also extruded in the same condition, as a reference.

### 2.5. Test Sample Preparation

The extruded composite materials were ground into small particles by a grinder for further use. The tensile test specimens (dog-bone-shaped, ISO 527, type 1A) and UV weathering specimens (80 mm × 10 mm × 4 mm) were carried out by a micro-compounder (Micro 15, DSM Xplore, Sittard, The Netherlands) and a micro-injection molder (Xplore, Sittard, The Netherlands).

### 2.6. Characterization

A UV accelerated weathering test was carried out by a QUV accelerated weathering tester (SOLAR EYE, Q-Panel Lab Products, Saarbrucken, Germany). The specimens were exposed under continuous irradiation with an irradiance intensity of approximately 0.68 W/m^2^/nm at λ = 340 nm for 168, 336 at 60 °C. The anti-UV properties of tannin, crosslinked tannin, lignin, and crosslinked lignin were evaluated by the changes in the mechanical property (specimens under 168 h UV exposure, 3 repetitions), surface morphology, surface chemical property, rheological behavior, and crystallinity. 

The tensile test was molded by a micro-injection molder (Xplore, Sittard, the Netherlands) and performed at room temperature with standard dog-bone shaped (ISO 527, type 1A) tensile test specimens. The Instron tensile testing machine (model 5569) was equipped with a 50 kN load cell, operated according to EN ISO 527:1996 with a 10 mm/min cross-head speed. Four repetitions of unweathering samples and three repetitions of weathering samples were calculated in the average value. 

The rheological property was performed by using a rheometer (TA instrument, G2 ARES, New Castle, DE, USA) with parallel-plate geometry (25 mm diameter, 1 mm gap) with the protection of nitrogen under 180 °C. Frequency sweeps between 0.1 to 100 rad/s were carried out at 10% strain in the linear viscoelastic region (LVE). The samples for rheometer were cut from tested tensile specimens and weathered specimens.

The differential scanning calorimetry (DSC, METTLER TOLEDO, Columbus, OH, USA) was employed to measure the melting temperature (*T*_m_) and crystallinity temperature (*T*_c_) of samples in the second heat-cool-heat circle with a scan rate of 10 °C/min within the temperature range of −50 to 220 °C. The measurements were using aluminum crucibles with a total sample weight 3–4 mg under a nitrogen atmosphere (50 mL/min). Values for melting temperatures (*T*_m_) and enthalpy of melting (*H*_m_) were analyzed by STARe evaluation software (version 10.0, METTLER TOLEDO, Columbus, OH, USA). The percentage of crystallinity *X*_c_ was estimated by the following equation:

(1) $$X_{c}\left(\%\right)=\frac{100\times H_{m}}{\Delta H_{m}^{0}\times w}$$

where *X*_c_ is the crystallinity (%), $\Delta H_{m}^{0}$ is the enthalpy of melting 100% crystallized PP, which is equal to 207 J/g [45], *H*_m_ is the enthalpy required for melting each sample, and w is the weight fraction of PP in blends.

The thermal degradation study was carried out in a thermogravimetric analysis (TGA) instrument (METTLER TOLEDO, Columbus, OH, USA). All samples (6–8 mg) were scanned in the range of 30–600 °C with a heating rate 10 °C/min in an air atmosphere (50 mL/min). The obtained data were analyzed by STARe evaluation software (version 10.0, METTLER TOLEDO, Columbus, OH, USA).

The morphology of fracture surface and UV exposed surface were characterized by a scanning electron microscopy (SEM, JSM-6490LV, Tokyo, JAPAN) with an acceleration voltage of 5 kV. All the samples were sputter coated with a thin layer of carbon.

The Fourier transform infrared (FT-IR) spectra of the non-weathered and weathered surface of specimens measured by a NICOLET 6700 FT-IR (Waltham, MA, USA) spectrometer attenuated total reflection (ATR) mode for 16 scans in the range 4000–650 cm^−1^ with a resolution of 4 cm^−1^. The curves were normalized, and peaks were analyzed without smoothing the data. The degradation of composites was characterized by carbonyl index(CI) [48]. The calculation equation as follows:

(2) $$CI=\frac{A_{c}}{A_{r}}$$

where *A*_c_ is the area of the carbonyl absorption band (1670–1820 cm^−1^, C=O). Ar is the area of C–H stretch from CH_3_ (2760–3020 cm^−1^). The latter one was used as a reference band because it was minimally affected by UV irradiation. For a more precise evaluation, the calculated carbonyl area Ac of each sample has been subtracted from the area before aging, considering that tannin and lignin have carbonyl groups in their structure. 

## 3. Results and Discussion

### 3.1. The Effects of Native Tannin/Lignin and Vulcanized Tannin/Lignin on PP Matrix

#### 3.1.1. Mechanical, Crystalline, and Thermal Properties

**Figure 2:**
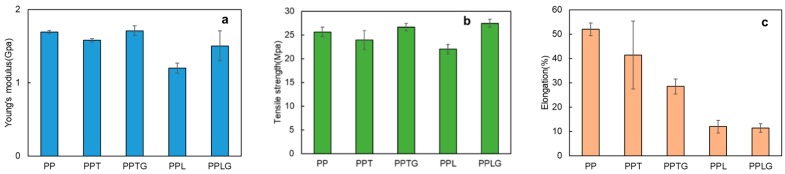
The tensile property of pure polypropylene (PP), PP/polyphenol blends, and PP/crosslinked polyphenol blends: (**a**) Young’s modulus; (**b**) Tensile strength; (**c**) Elongation.

The tensile test results of PP/tannin blend (PPT), PP/tannin-glyoxal blend (PPTG), PP/lignin blend (L), and PP/lignin-glyoxal blend (PPLG) are shown in Figure 2. As can be seen from the bar chart, a clear decrease of Young’s moduli can be found for PPT and especially for PPL. However, PPTG and PPLG display a general increasing trend compared with PPT and PPL. Young’s modulus is the stiffness of material at the elastic stage of a tensile test. For thermoplastic composite materials, this property can be readily improved by adding rigid fillers because the rigidity of fillers is generally much higher than that of PP matrix [49]. Therefore, a further crosslinked process happens to both pre-reacted tannin and lignin during extrusion process producing rigid fillers. This can explain the improvement of Young’s moduli. 

**Figure 3:**
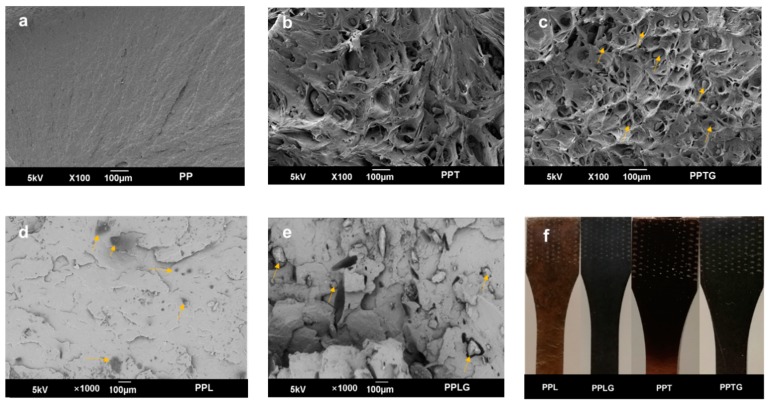
SEM images of fractured surfaces of pure PP (**a**), PP/polyphenol blends (**b**,**d**), and PP/crosslinked polyphenol blends (**c**,**e**) tested by tensile measurements; images of tested dog-bond specimens (**f**).

A lower Young’s modulus was observed for PPL when compared to PPT. This result can be explained by worse lignin particles dispersion in the PP matrix (Figure 3f). According to the study of Bozsódi [50], particle size is a very important factor that can significantly affect the mechanical properties of PP/lignin blends. At the same mixing condition, the utilization of coupling agent can prevent lignin from agglomeration, thus, reducing particle size and improving the miscibility. In our case, glyoxal can reduce the coalescence of lignin oligomer, thus, smaller lignin particle sizes resulting in a better dispersion (Figure 3f). Therefore, the dispersion degree of polyphenols in the polymer matrix can be improved through the dynamic vulcanization process; this can be a potential pathway to process thermoplastic/polyphenols composites, especially for polypropylene/lignin blends.

Similarly, the tensile strength of PP blended with crosslinked tannins (PPTG) and crosslinked lignin (PPLG) display higher tensile strength compared with that blended with PPT, PPL, and even with PP. Tensile strength is the maximum stress a material can sustain under uniaxial tensile loading. For composite materials, tensile strength depends on the stress transfer capacity between fillers and polymer matrix, which is significantly affected by particle size, particle distribution, and particle/matrix interfacial strength [50]. Considering the structure of PP is a very polar polymer that contains only carbon and hydrogen atoms and is capable of forming very weak dispersion interactions, the blends of PP and hydrophilic tannin and lignin is expected to form poor interface adhesion, resulting in a poor tensile strength. However, the increase of tensile strength of those blends with crosslinked tannins and lignin confirms better compatibility with PP matrix. Because the dynamically vulcanized process reduces the hydrophilic characteristic of both tannin and lignin, those crosslinked tannin and lignin are easier to disperse in PP matrix during the extrusion process; thus, a good particle distribution attributes to a better stress transfer capacity between polyphenols and PP matrix. In Figure 2c, a general decrease of elongation in all PP/polyphenols blends can be observed. The crosslinked tannin and lignin display high moduli and those rigid fillers would lend stiffness to the composite material [51]. 

A separation of filler and PP matrix can be observed from their SEM micrographs in Figure 3. Unlike the relatively smooth surface of native PP, plastic matrix pulls are generally observed. As commonly observed in filled PP composites, PP matrix deformed independently due to the weak filler-matrix adhesion between polyphenols and PP matrix until the polyphenol particles restrict the deformation. In PP/tannin blends, the identification of tannin particles in tensile fracture surface is difficult because of their low particle size. However, the crosslinked tannin particles in different sizes and irregular shapes particles are clearly found in Figure 3c (marked in yellow narrow), which confirmed the formation of tannin thermoset particles. Similarly, results can be found in PP blends with crosslinked lignin (Figure 3e). Unlike PP/tannin blends, obvious agglomeration of lignin can be found in both SEM micrographs (Figure 3d) and the image of tested specimen (Figure 3f); this result confirmed that lignin agglomeration easily occurs in the melt blending process without modification. Therefore, it can be concluded that the dispersion degree can be the main reason for the improvement of the tensile property. 

**Figure 4:**
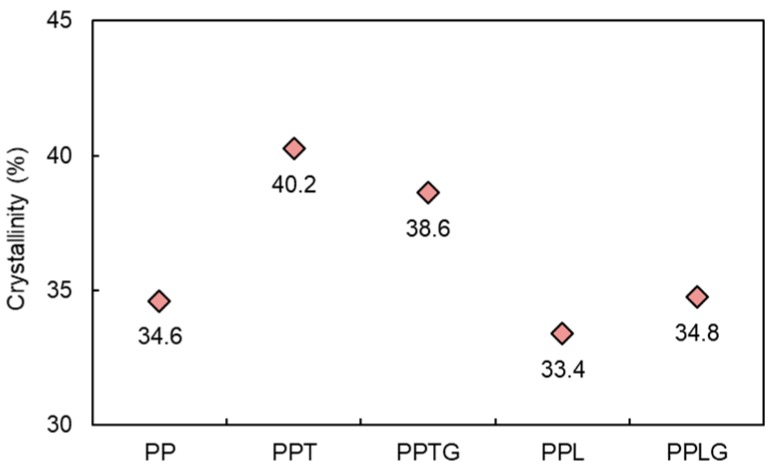
The crystallinity index of PP, PP/polyphenol blends, and PP/crosslinked polyphenol blends.

The addition of filler commonly affects the crystallinity of the polymer matrix, especially in PP, a semi-crystalline polymer. Figure 4 presents the crystallinity of PP/polyphenol blends (PPT, PPL), and PP/crosslinked polyphenol blends (PPTG, PPLG). Various fillers [52,53,54], including thermoset resin [55], have been reported to provide positive effects on the crystallinity of PP. Similar results can be found in our recent publication regarding tannin-hexamine thermoset particle-filled PP [45]. In this study, both PPT and PPTG present higher crystallinity compared with PP. This result is in accordance with the improvement of tensile properties previously observed (Figure 2). The higher crystallinity of PPT compared to PPTG can be rationalized by the smaller particle sizes of the non-cross-linked tannins observed in SEM images (Figure 3). In contrast with previous reports [48], the addition of lignin reduced the crystallinity of PP matrix. This can be explained by the agglomeration of lignin particles previously observed. With reducing the hydrophilic property of lignin through the dynamic crosslinked process, the better particles dispersion resulted in an increase in the number of nucleating agents and a further increase in the crystallization rate of PP in the blends. 

**Figure 5:**
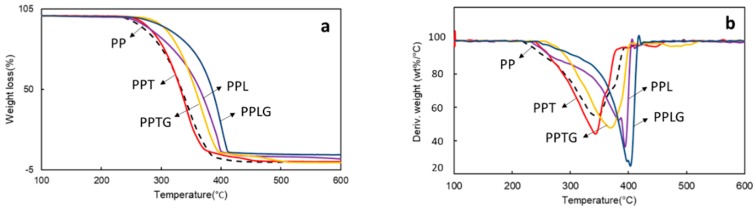
Thermal behaviors of PP, PP/polyphenol blends, and PP/crosslinked polyphenol blends: (**a**) TGA curves; (**b**) DTG curves.

The effects of native and crosslinked tannin/lignin on the thermal behavior of the PP matrix were determined by thermogravimetric analyses in an air atmosphere. Their weight loss as a function of temperature and derivative thermo-gravimetric (DTG) data are plotted in Figure 5. All samples degraded in a one-step process, from 220 to 410 °C, as can be concluded by the presence of only one peak in the DTG curves. Pure PP degraded completely without any char while the PP/polyphenol blends exhibited some char at 420 °C. This behavior leads to the formation of a protective surface shield and their interactions between PP, contributing to the considerable heat resistance [56]. However, PP/polyphenol blends represent noticeable differences in the thermo-gravimetric curves. The PP/lignin blends have a better heat resistance compared with PP/tannin blends. The different char forming capacity can be an explanation. According to the report of Sonnier [7], the char yield of lignin was 57 wt % while that of tannin was generally 30 wt %. Another possible reason could be related to molecular weight differences. According to the published articles, the average molecular weight of mimosa tannin (molecular weight distribution ranged from 375 to 2333 Da [46]) was generally much lower than that of Kraft lignin (*M*_n_ = 6772 g/mol [47], *M*_n_ = 5202 g/mol [57]). On this basis, the large molecular weight of lignin under thermo-degradation process results in the breakdown of linkages producing reactive and unstable lignin-derived fragments. These fragments may further react through rearrangement, electron abstraction, or radical–radical interactions, to effectively delay PP from degradation [58]. However, a similar thermo-degradation and interaction may occur in PPTG and PPLG blends, considering that un-crosslinked tannin/lignin existed because of their stereo-chemical structure and the limited resident time in the extruder (approximately 2 min 30 s). Besides, the pre-reaction of tannin and lignin were performed in alkaline condition (see Material and Methods section) [14,23], which resulted in linkage breakdown of polymers, increase in the phenolic content, and promotion of the antioxidant property [21]. The phenolics basic depolymerization could improve the radical capturing capacity of crosslinked tannin and lignin, resulting in better heat resistant of PPTG and PPLG.

#### 3.1.2. Rheological Behavior

**Figure 6:**
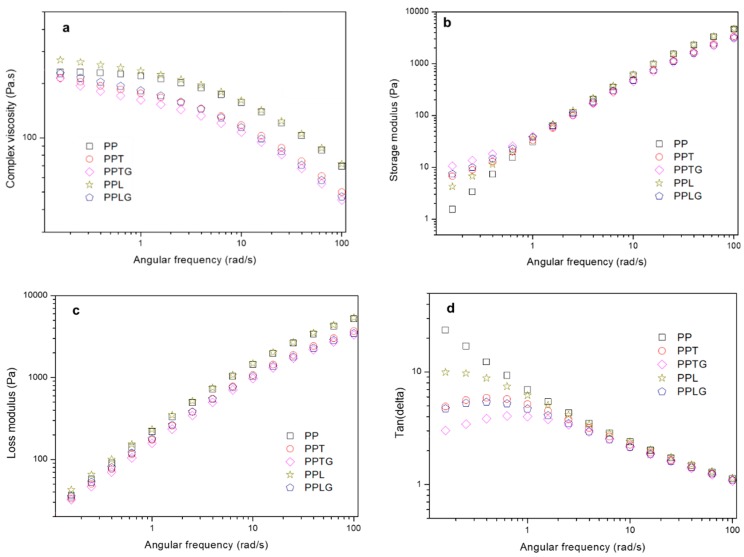
Rheological behaviors of PP, PP/polyphenol blends, and PP/crosslinked polyphenol blends: (**a**) complex viscosity; (**b**) storage modulus; (**c**) loss modulus; (**d**) tan(delta).

Figure 6a–d presents the rheological behaviors including complex viscosity (ƞ*), storage modulus (*G*’), loss modulus (*G*’’), and tan(delta) of PP, PP/polyphenol blends (PPT and PPL), and PP/crosslinked polyphenol blends (PPTG and PPLG). In particulate-filled polymers, melt rheological properties provide an efficient and reliable way to investigate filler properties and dispersion quality [59,60,61]. Particulate dispersion, which is related to particulate characteristics (surface property, shape, and size), particle content, and particle–matrix interaction, can significantly influence the viscoelastic response of filled polymers. As presented in Figure 6a, complex viscosity in all curves decrease as a function of angular frequency, exhibiting a typical shear-thinning behavior. As is reported by Hornsby [62], surface treatment organic fillers normally reduce the complex viscosity due to the decrease of particle agglomeration. Similarly, a decrease of complex viscosity is generally found in all curves (except PPL) compared with PP, indicating a better dispersion of fillers because the crosslinking process reduces the hydrophilicity of tannin and lignin. For those uncrosslinked tannin and lignin, PPL displayed an increase of complex viscosity in low-frequency region mainly caused by the agglomeration of lignin, which can be clearly observed in the image of specimen (Figure 3f). While it is hardly found, the agglomeration of tannin in the PP matrix may be due to the lower molecular weight of tannin compared with lignin. Besides, as observed from SEM microscopy (Figure 3), bigger particles size of lignin tends to effectively restrict the PP chain movements. 

Figure 6b,c presents the storage moduli and loss moduli as a function of the frequency of all tested samples. From those figures, a generally higher value of loss moduli than that of storage moduli can be observed. For loss moduli, there is no significant difference in all samples, however, an increase of storage moduli can be found in all filled PP samples at low frequency, suggesting that the presence of both native and crosslinked tannin/lignin limits the mobility of polymer chain, reinforcing the internal network structure. However, both PPTG and PPLG have higher storage moduli compared with PPT and PPL; this confirms the formation of thermoset particles in the PP matrix. Figure 6d, tan (delta), which is defined as the ratio of loss moduli and storage moduli, is plotted as a function of angular frequency. The tan (delta) values of all tested samples are above 1 and this implies that they are viscous-like materials. With the addition of tannin or lignin, tan (delta) is clearly observed to be lower than PP in the low-frequency region. This suggests that the mobility of the polymer chains is limited by polyphenols; thus, those filled samples show more elastic behaviors. Particularly, it is worth noting that PPTG and PPLG present a lower tan(delta) compared with PPT and PPL, indicating that crosslinked tannin or lignin more significantly restrict the movement of PP polymer chain. This can be explained by the formation and better dispersion of rigid thermoset particle through the dynamic extrusion.

### 3.2. Characterization of UV Protection Property of Native Tannin/Lignin and Vulcanized Tannin/Lignin on PP Matrix

**Figure 7:**
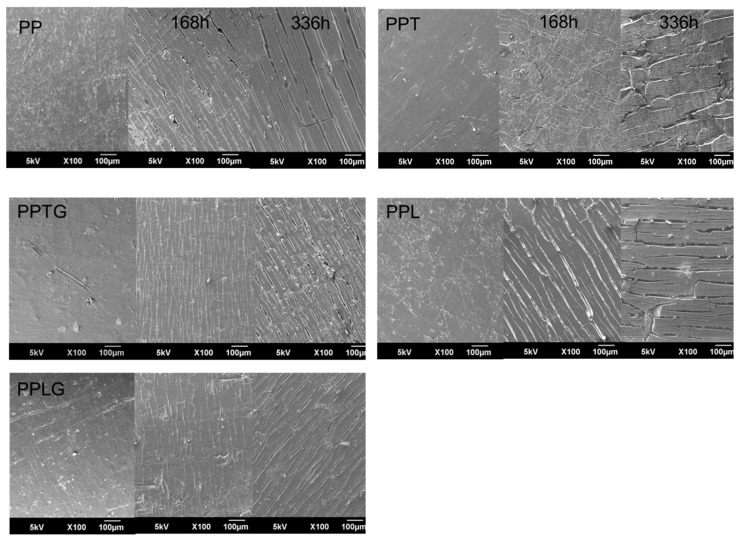
SEM microscopy of PP and PP/polyphenol blends over exposure time.

The photo-degradation of PP commonly starts from the surface, results in surface cracking, and ultimately lower mechanical properties. Thus, surface morphology is an efficient pathway to evaluate the degree of degradation. The surface exposed at 0, 168, and 336 h UV irradiate of PP, PP/polyphenol blends, and PP/crosslinked polyphenol blends were characterized by SEM and are presented in Figure 7. The micrographs before UV weathering with no visual cracks can be observed at the surface. However, after UV weathering, all exposed materials present strong visual surface variations. In the micrographs with 168 h exposure, PPTG and PPLG, which have similar surface morphology, exhibit micro-cracks at the surface, whereas large and long cracks were observed in PP, PPT, and PPL. More obvious differences can be found in the micrographs with 336 h exposure. At the surface of PP, PPT, and PPL, large and deep cracks are clearly observed whereas only some large cracks mixed with tiny cracks occurred at the surface of PPTG and PPLG. Besides, the morphology of PPL was similar to PP and severer cracks can be found compare with PPT, which might be caused by the worse dispersion of lignin in the PP matrix. All the mentioned phenomena were similar to their thermal behaviors because either photo-degradation or thermo-degradation mechanisms of PP are due to the appearance of free-radical species or peroxides in the polymer initiated by UV irradiation or temperature. In conclusion, both native or crosslinked tannin/lignin can prevent or interrupt the degradation processes. But crosslinked tannin or lignin appears to more significantly inhibit oxidation or inhibit reactions promoted by oxygen or peroxides.

**Figure 8:**
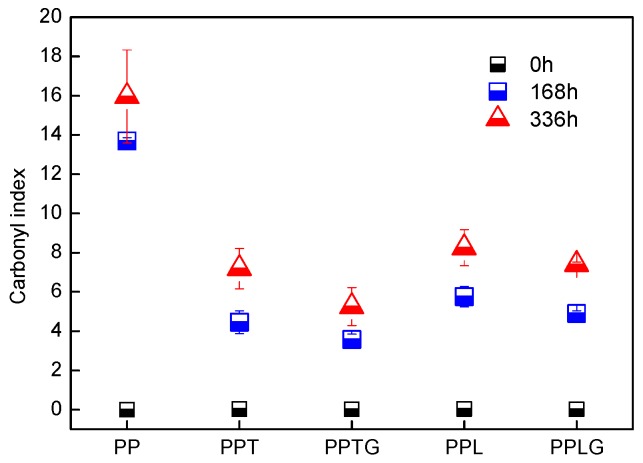
Carbonyl index of PP and PP/polyphenol blends over exposure time.

Figure 8 presents the calculated carbonyl index of PP and PP/polyphenol blends at 168 and 336 h exposure. The carbonyl indexes were established from FTIR spectra (see Materials and Methods section). As previously proposed, the carbonyl groups concentration exhibits a close correlation with the degradation degree since carboxylic acids are produced during the oxidation process [63,64]. As expected, all the samples show, on the exposed surface, an increasing carbonyl index with UV exposure time. However, compared to neat PP, the increase of carbonyl index for PP/polyphenol blends was lower, attesting the fact that polyphenols can capture free radicals and inhibit the oxidative reaction. Moreover, in accordance with the result from SEM micrographs (Figure 7), PPT and PPL present lower carbonyl indexes compared with PPTG and PPLG, respectively. This confirms that dynamic curing tannin/ lignin improved their antioxidant capacity. 

**Figure 9:**
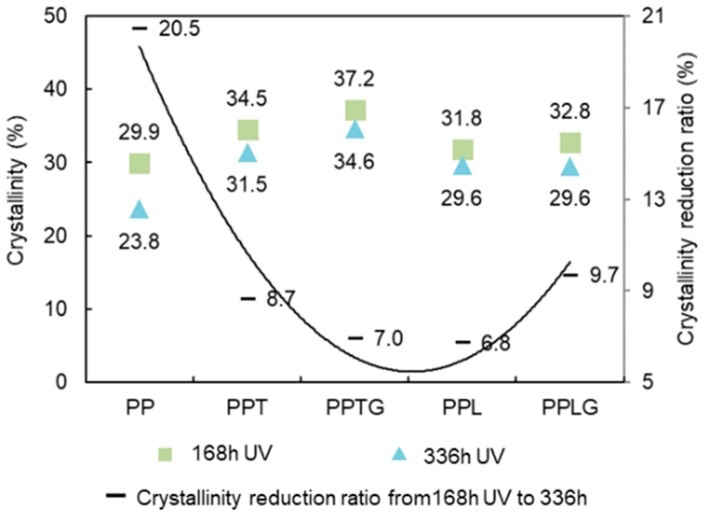
The crystallinity of PP and PP/polyphenol blends over exposure time.

Figure 9 displays the crystallinity of PP and PP/polyphenols blends over UV exposure. The reduction ratio of their crystallinities from 168 to 336 h is plotted as well. A drop in crystallinity can be observed after UV exposure in all samples because of the chain scission. More importantly, between 168 and 336 h of UV weathering, a decrease of crystallinity clearly was observed for neat PP (20.5%) compared to phenolic-based composites (<10%). Upon exposure to air and UV, photo-oxidation occurs by the appearance of free radicals. This oxidation is a circular, self-propagating process that, unless intruded on by an antioxidant, progressively leads to increasing the breaking and shortening of the polymer chain [10]. Therefore, the presence of tannin/lignin with the radical scavenge capacity inhibit chain scission of the polymer chain, reducing the decrease of crystallinity. 

**Figure 10:**
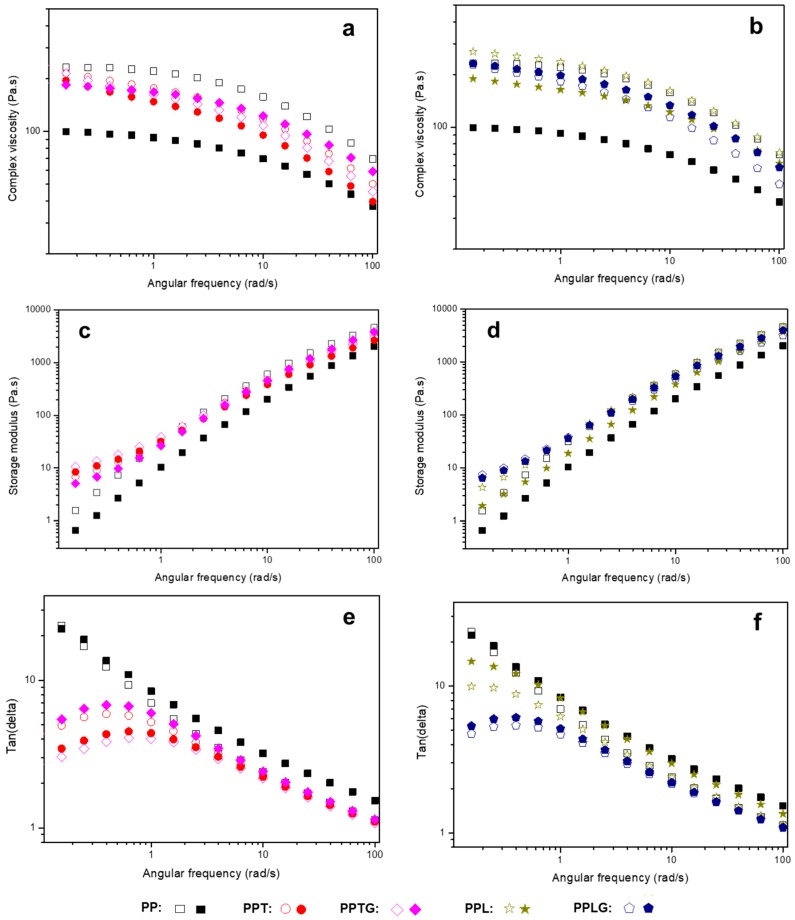
A comparison of rheological behavior of PP, PP/tannin blends (left), and PP/lignin blends (right) at 168 h UV weathering: (**a**,**b**) complex viscosity; (**c**,**d**) storage modulus; (**e**,**f**) tan(delta).

Figure 10 displays complex viscosity, storage modulus, and tan (delta) as a function of the angular frequency of PP and all PP/polyphenol blends at 168 h UV exposure. Compared to PP/polyphenol blends, a sharp drop of viscosity can be observed for neat PP after weathering, explained by the oxidative reduction in molecular weight. In addition to the inhibition of degradation, tannin and lignin might also contribute to the entanglement of degraded PP chain, thus limiting the melting flow of polymer chains [45]. As a result, for PPTG and PPLG, an increase in viscosity can be observed at high frequency because of the good dispersion of crosslinked tannin or lignin. In Figure 10c,e, we observed comparable results to those described for unweathering samples (Figure 6). This confirms their reinforcing role in PP matrix even after UV weathering. However, some decreases can be observed in storage modulus and tan (delta), suggesting that weathering samples appeared to have more viscous behaviors and the polymer degradation increased the melting flow.

**Figure 11:**
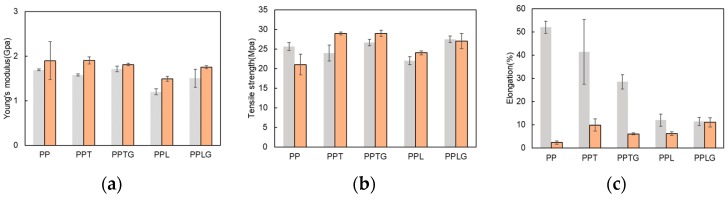
Young’s modulus, tensile strength, and elongation of PP and PP/polyphenol blends after weathering: (**a**) Young’s modulus; (**b**) Tensile strength; (**c**) Elongation. Grey-before weathering; orange-168 h UV weathering

In Figure 11, a rise of Young’s moduli can be found in all samples after 168 h of UV exposure at 60 °C, which is generally found in PP/polyphenol blends [65,66]. This can be explained by chain scission and the crosslinked phenomenon of molecules chain during accelerated weathering and chain movement obstacle caused by polyphenols. UV weathering did not significantly deteriorate the tensile strength of PP composite containing polyphenols. According to the work of Stark [67], serious mechanical loss of polyolefin occurs in sufficient photodegradation, accompanied by serious severe surface cracks and crystallinity decrease. PP/polyphenol blends have been proven to undergo less photodegradation with PP (see Figure 7, Figure 8, Figure 9 and Figure 10 and related discussion). Therefore, PP/polyphenols blends display comparable or increasing tensile strength after UV exposition with consideration of entanglement of polyphenols and PP polymer. A decrease in elongation can be found in all samples. The elongation of polyolefin has been found to be a more sensitive measurement of the extent of degradation than the tensile strength because the elongation at break of PP is dependent on its crystallinity [68]. In our case and in accordance with Lv and coworkers [64], the changes of crystallinity (Figure 4 and Figure 9) are in agreement with the elongation decrease. In addition, the entanglement of polyphenols and PP polymer chain can be another reason for less drop in elongation. This confirms the UV protection capacity of both native and crosslinked tannin/lignin. 

## 4. Conclusions

In this study, dynamic vulcanization has been successfully applied to PP/tannin and PP/lignin blends. The influence of vulcanized tannin/lignin on mechanical property, crystallinity, thermal stability, rheology behavior, as well as UV protection property of PP matrix was investigated. This extrusion process leads to tannin and lignin crosslinking into rigid thermoset particles, contributing to the improvement of Young’s modulus of the composite. After vulcanized extrusion, the hydrophilicity of tannin and lignin were reduced, thus increasing the compatibility in the PP matrix. Besides, tannin and vulcanized tannin can act as nucleating agents of PP because of their particle size. Vulcanized tannin/lignin have a better performance on the thermal stability of PP compared with native tannin/lignin, especially for PP-vulcanized lignin. From the rheological data, vulcanized tannin/lignin displayed a reinforce function and confirmed their better dispersion capacity in the PP matrix compared with native tannin/lignin. Furthermore, vulcanized tannin/lignin present better UV protective performance, demonstrated by fewer changes on surface morphology, carbonyl index, crystallinity, viscosity, and tensile property. 

## Figures and Tables

**Table 1:** The parameters of spray-drying.

Inlet Temperature	Aspirator	Pump Rate	Nozzle Cleaner	Feed Switch Valve	Flow Meter (mm)
150 °C	100%	10%	6	1	40
